# Inhibiting HSP90 changes the expression pattern of PINK1 and BNIP3 and induces oxidative stress in colon cancer cells

**DOI:** 10.1007/s11033-025-10303-x

**Published:** 2025-02-08

**Authors:** Ejlal Abu-El-Rub, Ayman Alzu’bi, Fatimah A. Almahasneh, Ramada R. Khaswaneh, Rawan Almazari, Amani Kasasbeh, Ala A. Aldamen, Heba F. AI-jariri, Amal Alomari, Tuqa Yousef, Raed M. Al-Zoubi

**Affiliations:** 1 Department of Basic Medical Sciences, Faculty of Medicine, Yarmouk University, Irbid, 211-63 Jordan; 2 Surgical Research Section, Department of Surgery, Hamad Medical Corporation, Doha, Qatar; 3 Department of Biomedical Sciences, QU-Health, College of Health Sciences, Qatar University, 2713 Doha, Qatar; 4 Department of Chemistry, Jordan University of Science and Technology, P.O.Box 3030, Irbid, 22110 Jordan

**Keywords:** RKO cells, HSP90, PINK1, BNIP3, Oxidative stress, Apoptosis

## Abstract

**Background:**

Cancer cells can modulate the expression of many proteins that are essential for supporting their uncontrolled proliferation. Heat shock protein 90 (HSP90) is ubiquitously expressed in most cell types and participates in controlling many survival pathways. Cancer cells utilize HSP90 in order to prolong their survival, thus they tend to overexpress it. Based on its importance for cancer cells, we aim to investigate the molecular mechanisms that link HSP90 inhibition in colon cancer cells with oxidative stress and mitochondrial stress—related regulators.

**Materials and methods:**

We used RKO colon cancer cells, blocking HSP90 with the inhibitor AT13387 and HSP90 siRNA. Cell proliferation and apoptosis were measured via CCK8 ELISA and Fluorescent Apoptosis Assays. Western blotting and immunocytochemistry assessed oxidative and mitochondrial stress markers BNIP3, PINK1, GP91/NOX2, and IRE1α in treated cells.

**Results:**

Our findings reveal that inhibiting HSP90 with AT13387 reduces RKO cell viability by suppressing proliferation and enhancing Annexin-V expression, indicative of increased apoptosis. This rise in apoptosis is associated with PINK1 downregulation and BNIP3 upregulation, markers of mitochondrial dysfunction and oxidative stress, respectively. Additionally, AT13387 treatment elevated the protein level of GP91, a marker of oxidative stress, and IRE1α, a marker of ER stress. Similarly, genetic knockdown of HSP90 in RKO cells produced comparable effects, including reduced cell survival and a decreased PINK1/BNIP3 ratio.

**Conclusion:**

Targeting HSP90 in colon cancer cells disrupts their survival by decreasing PINK1 and increasing BNIP3, which activates oxidative and endoplasmic reticulum stress, ultimately triggering apoptosis.

**Supplementary Information:**

The online version contains supplementary material available at 10.1007/s11033-025-10303-x.

## Introduction

Colorectal cancer is considered a leading cause of cancer-related morbidity and mortality, with millions of new cases diagnosed each year [1]. Surgery, chemotherapy and radiotherapy have traditionally been the first choices for the treatment of cancer, but although these methods have been proven to save thousands of lives, they often significantly affect quality of life[2]. The availability of alternative options alongside the classic approaches offered new treatment opportunities to patients who were unable to pursue the classic therapies due to added toxicities, treatment resistance or comorbidities.

Heat shock protein 90 (HSP90) is a highly conserved molecular chaperone that catalyzes protein folding, stabilization, and degradation, assisting in structural integrity and proper regulation of proteins [3]. HSP90 serves as a central hub in the regulation of various signaling pathways crucial for cell survival, proliferation, and the transition between sequential stages of cell cycle [4]. Mitochondrial intactness is considered crucial for cancer cells to evade cell death. Cancer cells, including colon cancer, have high tendency to increase the rate of mitochondrial biogenesis and the level of mitochondrial quality controllers. These modulations occurred in cancer cells are fundamental for maintaining the mitochondrial integrity and prevent the production of undesirable level of Reactive oxygen species (ROS) which can mediate that activation of other intracellular pathways, including inflammation and apoptosis [5]. HSP90 is considered important for maintaining proper mitochondrial dynamics in many cells [6]. Previous studies have demonstrated that HSP90 exhibits high activity levels within tumor tissues, including colon cancer, allowing cancer cells to evade stress-induced mechanisms of cell death and continue to proliferate and invade [7]. Given its central role in promoting colon cancer cell survival under stress conditions, such as hypoxia, HSP90 has emerged as an attractive therapeutic target in cancer treatment [8].

Inhibiting HSP90 in cancer cells has been purported to be an effective strategy in inciting cell death signaling pathways that can successfully curb their growth and proliferation. The exact downstream targets following HSP90 inhibition need thorough investigation. BCL2/adenovirus E1B 19 kDa protein-interacting protein 3 (BNIP3) plays a crucial role in the regulation of apoptosis, autophagy, and mitochondrial function [9]. BNIP3 modulates cancer cell survival by orchestrating cellular responses to stress conditions and inhibiting tumor growth and metastasis [10]. Interestingly, while BNIP3 levels are elevated in several cancer types [11], studies have reported a decrease in BNIP3 expression in colon cancer, which results in a failure of tumor cells to undergo cell death [12].Phosphatase and tensin homolog‐induced kinase 1 (PINK1) is a putative kinase that contributes to mitochondrial stabilization and quality control [13]. PINK1 was found to play a role in the pathophysiological processes in cancer cells, including cytoplasmic homeostasis, and cell survival and death [14]. The association between HSP90, BNIP3, and PINK1 has not been investigated thoroughly. In this study we explore the molecular mechanisms of HSP90 inhibition on colon cancer cells with regard to oxidative and mitochondrial stress—related regulators. This is expected to contribute to the development of effective targeted cancer therapies, broadening and improving the available therapeutic options for cancer, particularly resistant forms.

## Materials and methods

### Cell lines

The human RKO colon cancer cell line was provided from Prof Stephan Feller from the Institute of Molecular Medicine at Martin-Luther –University, Halle-Wittenberg, 06120, Halle, Germany. The normal cells used are HIEC-6 purchased from ATCC which are epithelial cells that was isolated from the small intestine. Cells were cultured in high glucose DMEM culture media supplemented with 10% FBS (Euroclone, Italy), 40 U/ml penicillin and 100 U/ml streptomycin at 37 °C in a humidified atmosphere containing 5% CO2 throughout the present study. Cells were grown to 70% confluency, detached, and counted to the start of each experiment.

### Reagents and chemicals

HSP90 inhibitor: AT13387 (GlpBio, USA). Antibodies used for Western blotting and Immunostaining: HSP90 (Santa Cruz Biotechnology, Cat # sc-13119), PINK1 (Santa Cruz Biotechnology, Cat # sc-518052), BNIP3 (R&D system, Cat# MAB41471), GP91/NOX2 (Santa Cruz Biotechnology, Cat # sc-74514), IRE1α (Santa Cruz Biotechnology, Cat # sc-390960), PERK (Santa Cruz Biotechnology, Cat # sc-377400), β-actin (Santa Cruz Biotechnology Cat # sc-47778 HRP), Goat anti-Mouse Alexa IgG Fluor 488 (Invitrogen, USA), Anti-mouse Alexa Fluor 647 (Invitrogen, USA), Mounting Medium With DAPI ( Abeam, UK), Ultra High Sensitivity ECL Kit ( GlpBio Cat# GK10008).

### HSP90 silencing and blocking

For the pharmacological inhibition of HSP90, RKO cells were treated with different doses of AT13387 (10 µm and 20 µm) for 24 h. For the genetic knockdown, we employed siRNA to knockdown HSP90 in RKO cells. We used HSP90 siRNA from Santa Cruz Biotechnology (sc-35608) and as a control (sc-37007). The siRNA used is a pool of 4 target-specific 19–25 nt siRNAs designed to knock down HSP90 gene expression. We used Lipofectamine 3000 from Invitrogen as a transfection reagent. Briefly, 10 µM stock solution of siRNA was prepared. 50,000 RKO cells were seeded per well on the coverslips in 12-well plate and 80 pmol of both targeting and non-targeting siRNA were added after incubating the siRNA with Liopfectamine 3000 for 40 min. This was followed by addition of siRNA-Lipofectamine complex to each well and incubation in the CO2 incubator for 24 h. Next day, the transfected cells were used to perform the subsequent experiments.

### CCK8 proliferation assay

The proliferation of RKO cells after 24 h of being treated with 10 µm and 20 µm AT13387 inhibitor was measured using the commercial kit (Cell Counting Kit-8 Kit, GLP BIO, Cat # GK10001). Briefly, RKO cells were seeded in 96 well plates (5 × 104 cells/well) and allowed to attach overnight. Then cells were treated with AT13387 for 24 h. Following that, we added 10 µl of CCK8 solution to each well and the plate was incubated in the CO2 incubator for 4 h. The absorbance values were taken at 450 nm using Cytation 5 system (BioTek, USA).

### Apoptosis assay

To detect apoptosis in untreated and treated RKO, we used RealTime-Glo™ Annexin V Apoptosis live assay (Promega, Cat# JA1011, Lot# 0000400486 following the manufacturer’s guidelines. Briefly, cells were seeded 1X105 cells/ well in 24 well plate and then treated with AT13387 for 24 h. The media were aspirated, and each well was washed with PBS followed by the addition of 100 µl of fresh high glucose medium having Annexin V-LgBiT, Annexin V-SmBiT, CaCl2, and Annexin V NanoBiT Substrate to each well. After 1 h incubation, the fluorescent images of green color which represented cells undergoing apoptosis were detected at GFP filter using Cytation 5 (BioTek, USA).

### The LIVE/DEAD® viability/cytotoxicity

LIVE/DEAD Viability/Cytotoxicity Kit (Invitrogen, Cat# L3224) was used to quickly discriminates life from dead cells following knocking down HSP90 in RKO cells. This assay detects live cells which represent green-fluorescent calcein-AM and dead cells which represent the red-fluorescent ethidium homodimer-1. Briefly, RKO cells were seeded at 50,000 cells /well in 24- well plate followed by using 80 pmol of HSP90 siRNA to transfect the seeded cells. After 24 h of transfection, 100 µl of the staining solution which contains calcein AM and 20 µL ethidium homodimer-1 in DPBS. The cells were incubated in the staining solution for 30 min followed by imaging the cells using Cytation 5 imaging system (BioTek, USA).

### Western blotting

The protein levels for HSP90, PINK1, BNIP3, GP91/NOX2, IRE1α, PERK, and β-actin were measured by Western blot. Briefly, normal cells and RKO were seeded in 10 cm cell culture dishes and allowed to attach overnight. Next, RKO cells were treated with AT13387 for 24 h. After treatment, cells were scraped, and protein was isolated using Radioimmunoprecipitation assay buffer (RIPA buffer) and Protease Inhibitor Cocktail. Total protein levels were measured using NanoDrop™ Lite Spectrophotometer, and 40 μg of protein was loaded onto SDS–PAGE. Following electrophoresis, proteins were transferred to PVDF membrane and were incubated with appropriate primary and secondary antibodies. The membranes were visualized using VILBER FUSION Gel Documentation System, and bands were quantified using ImageJ for densitometry.

### Immunocytochemistry

RKO cells were seeded onto sterile coverslips and allowed to grow till 60% confluency. The plated cells were fixed with 4% paraformaldehyde and permeabilized using 0.2% Triton X in PBS at RT. The cells were then stained with respective primary and secondary antibodies, Thereafter, the stained cells were preserved and counter-stained with Mounting Media having DAPI (4′,6-diamidino-2-phenylindole) for nuclei. The cells were imaged using Cytation 5 imaging system (BioTek, USA).

### Statistical analysis

Data was reported as mean ± SD. Comparison of data between multiple groups was performed using one-way analysis of variance (ANOVA) followed by Tukey’s post-hoc multiple comparison test, and analysis between two groups was made using Student’s t-test (two-tailed). Statistical significance is determined as P < 0.05. Each figure represents one of at least three independent quantifiable experiments.

## Results

### RKO Cells exhibited upregulation in the expression of HSP90 and PINK1

**Fig. 1:**
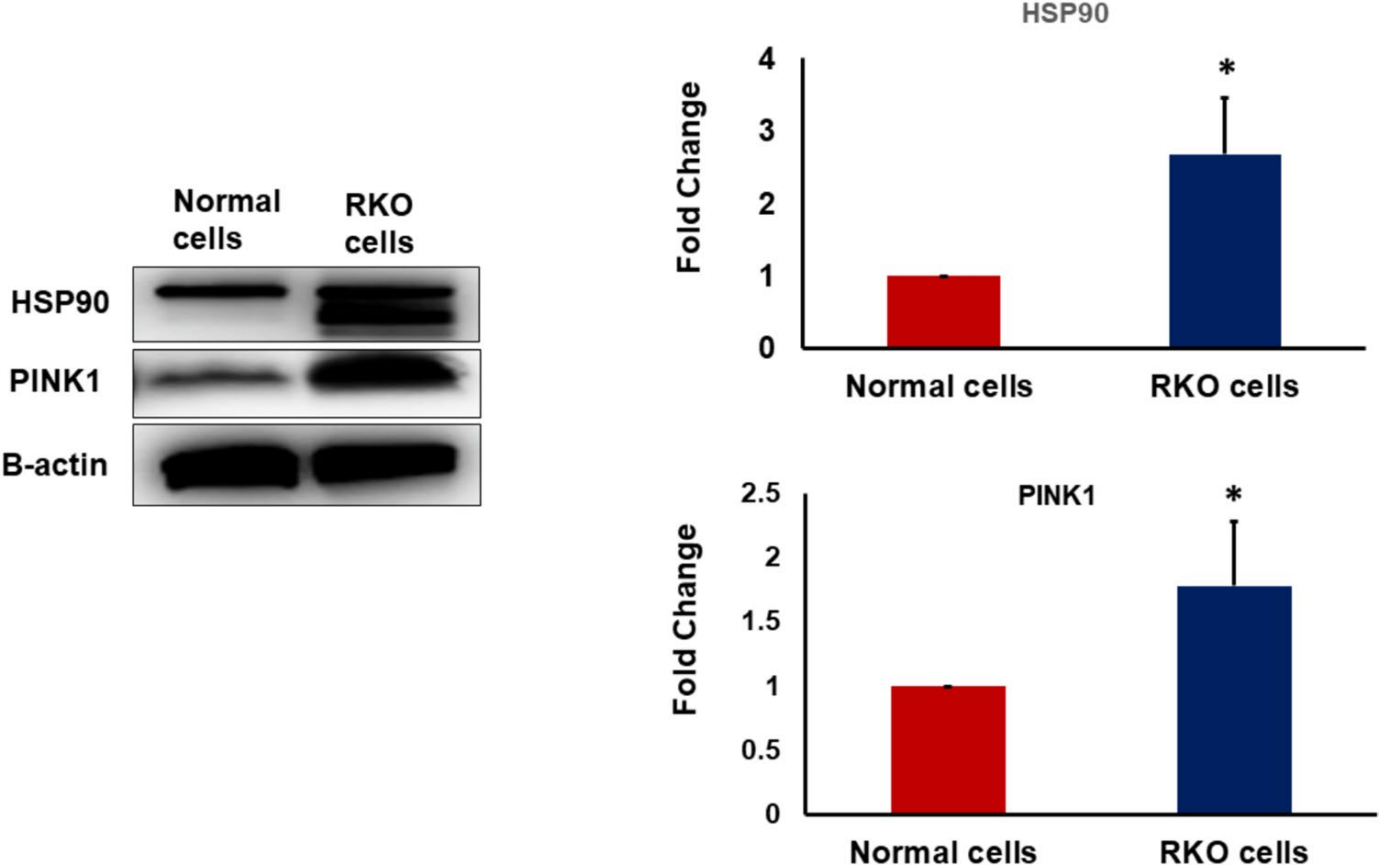
The protein expression of HSP90 and PINK1 in normal cells and RKO cancer cells. **A** Representative Western blot analysis of HSP90 and PINK1 in normal cells and RKO cancer cells. **B** The expression of HSP90 is significantly higher in RKO cells compared to the normal cells. **C** The expression of PINK1 is significantly higher in RKO cells compared to the normal cells. N = 4, *P < 0.05 is statistically significant compared to normal cells

Cancer cells have the ability to modify the expression of crucial molecules to support their survival and invasiveness. By acting as an important chaperone for controlling many cellular signaling pathways, we measured the protein level of HSP90 in normal epithelial cells derived from GI system and RKO cells. The protein level of HSP90 was found to be highly elevated in RKO cells compared to normal cells (Fig. 1A,B). The survival of cells whether they are normal or cancerous is supported and maintained by having functional mitochondria. As PINK1 plays a significant role in maintaining the intactness and functionality of mitochondria, we measured the protein level of PINK1 in normal and RKO cells and our results showed that PINK1 is expressively upregulated in RKO cells compared to normal cells (Figs. 1A, C).

### Inhibiting HSP90 reduced proliferation and increased apoptosis in RKO cells

**Fig. 2:**
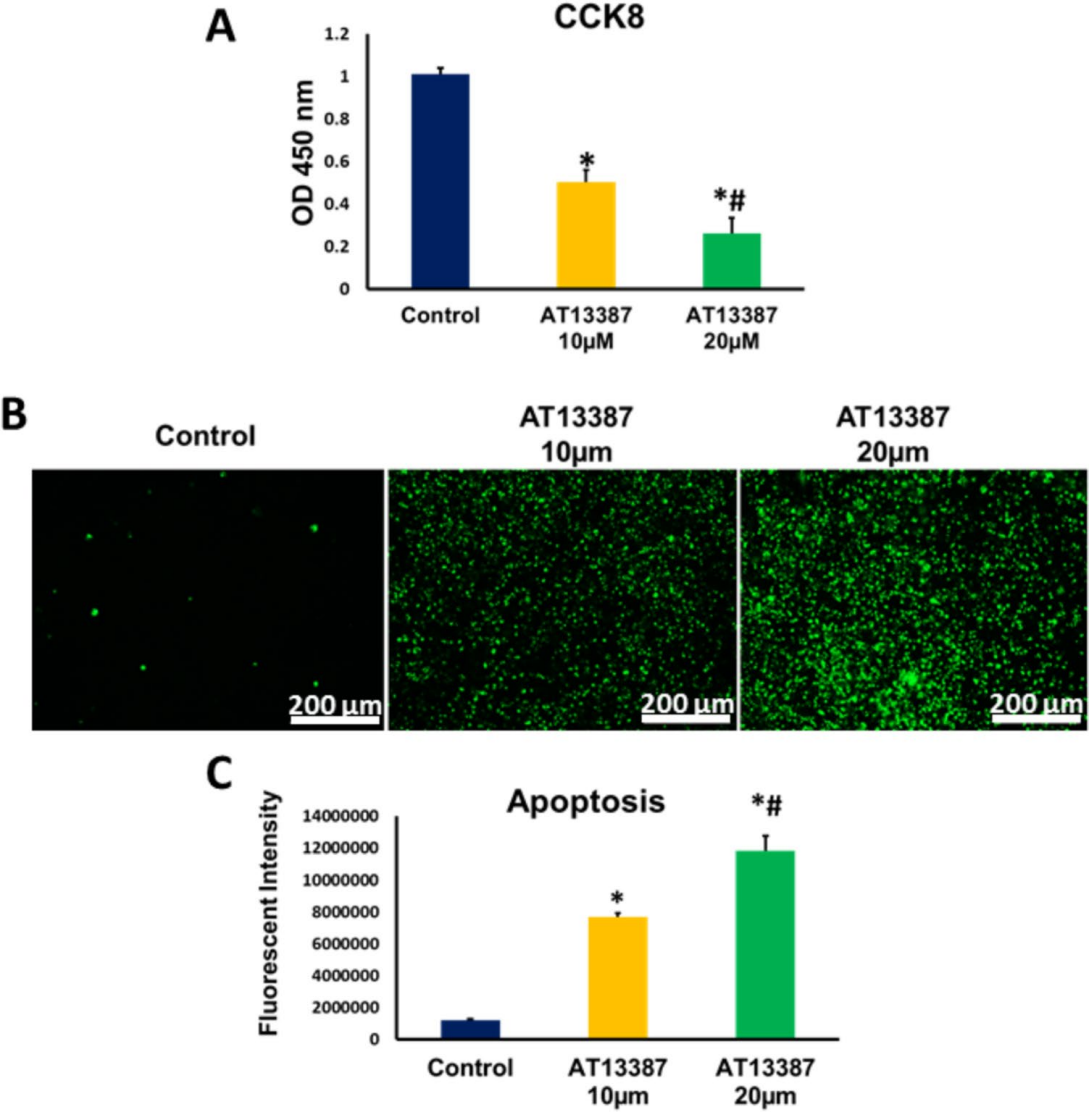
The proliferation and the level of apoptosis of RKO cells treated with different concentrations (10 and 20 μm) of AT13387. **A** The proliferation of RKO cells was (N = 6) significantly lower in AT13387-treated RKO cells compared to the control. The reduction in the proliferation demonstrated a dose-dependent effects of AT13387 inhibitor. **B** Fluorescent representative images showed the RKO cells (N = 4) undergoing apoptosis using the two doses of AT13387 along with a control group for comparison. Annexin V (green fluorescent fluorophore) was used to label the apoptotic cells. **C** A graph demonstrating the apoptosis intensity measured in Relative Luminescence Units (RLU) for the three groups. The Apoptosis of RKO cells treated with AT13387 was significantly higher than that of the control counterpart. The level of apoptosis exhibited a dose-dependent increase in AT13387-treated RKO cells. *P < 0.05 is statistically significant compared to control group. # p < 0.05 is statistically significant compared to 10 µm dose

RKO cells were treated with 10 µm and 20 µm of HSP90 inhibitor; AT13387, for 24 h, and the proliferation of RKO cells was then assessed using CCK8 Elisa kit. As shown in Fig. 2A, the proliferation of RKO cells was significantly decreased in 10 µm and 20 µm AT13387 -treated cells compared to control cells. A significantly lower proliferation was also observed in 20 µm AT13387 treated cells compared to 10 µm treated cells, indicating that HSP90 inhibition in RKO cells reduces the proliferation in a dose-related manner.

We next examined the effect of HSP90 inhibition on the level of apoptosis in RKO cells. Our results showed that HSP90 inhibition is significantly associated with dose-related increase in apoptosis in RKO cells (Figs. 2B, C).

### Inhibiting HSP90 altered the expression of PINK1 in RKO cells

**Fig. 3:**
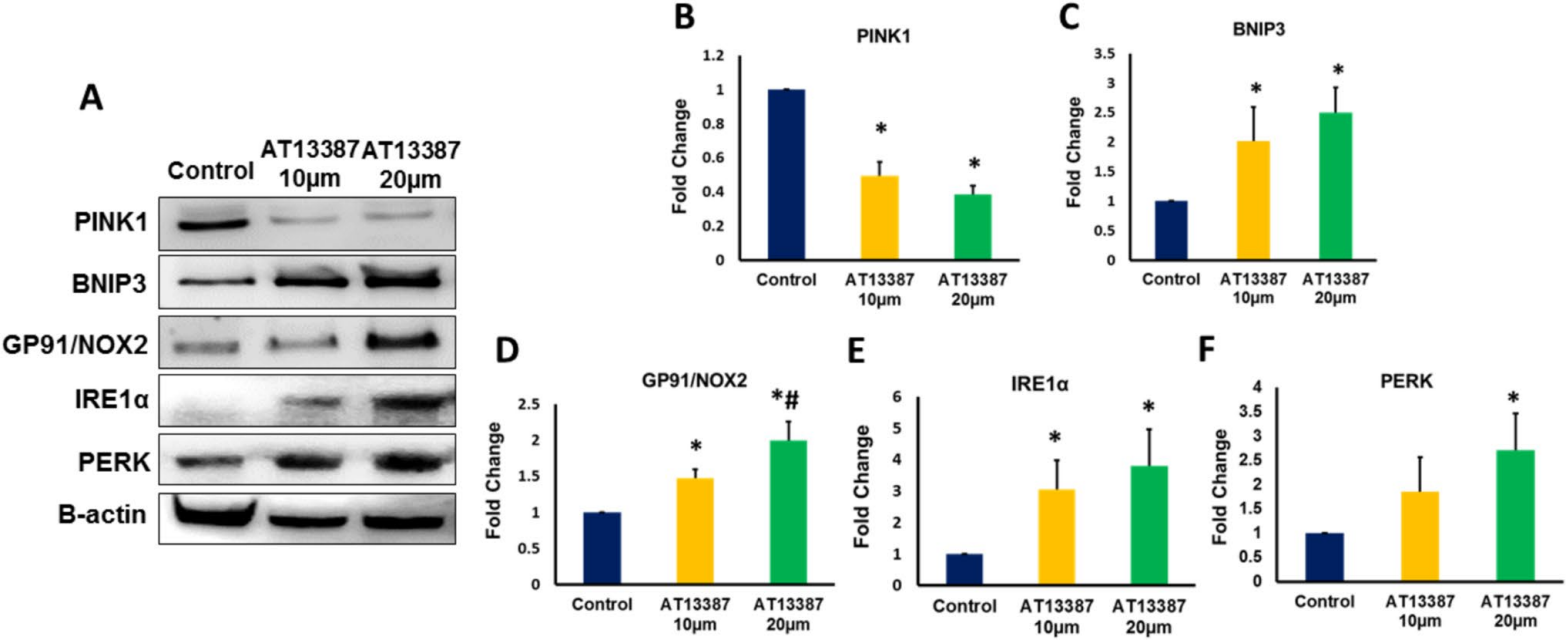
The protein expression of PINK1, BNIP3, GP91/NOX2, IRE1α, and PERK in RKO cells treated with AT13387 inhibitor. **A** Representative Western blot analysis of PINK1, BNIP3, GP91/NOX2, IRE1α, and PERK expression in AT13387-treated RKO cells. **B** The expression of PINK1 was significantly decreased in RKO cells treated with the two doses of AT13387 inhibitor (10 and 20 μm) compared to untreated control cells. **C** The expression of BNIP3 was significantly increased in RKO cells treated with the two doses of AT13387 compared to untreated control cells. **D** The protein expression of GP91/NOX2 was significantly increased in RKO cells treated with the two doses of AT13387 compared to untreated control cells. The protein level of GP91/NOX2 in RKO cells treated with 20 μm AT13387 showed a significantly higher expression of GP91/NOX2 compared to RKO cells treated with 10 μm AT13387 indicating a dose-dependent increase in its expression pattern. **E** The protein expression of IRE1α was significantly increased in RKO cells treated with the two doses of AT13387 compared to untreated control cells. **F** The protein expression of PERK was significantly increased in RKO cells treated with 20 μm AT13387 compared to untreated control cells. N = 4, *P < 0.05 is statistically significant compared to control group. # p < 0.05 is statistically significant compared to 10 µm dose

**Fig. 4:**
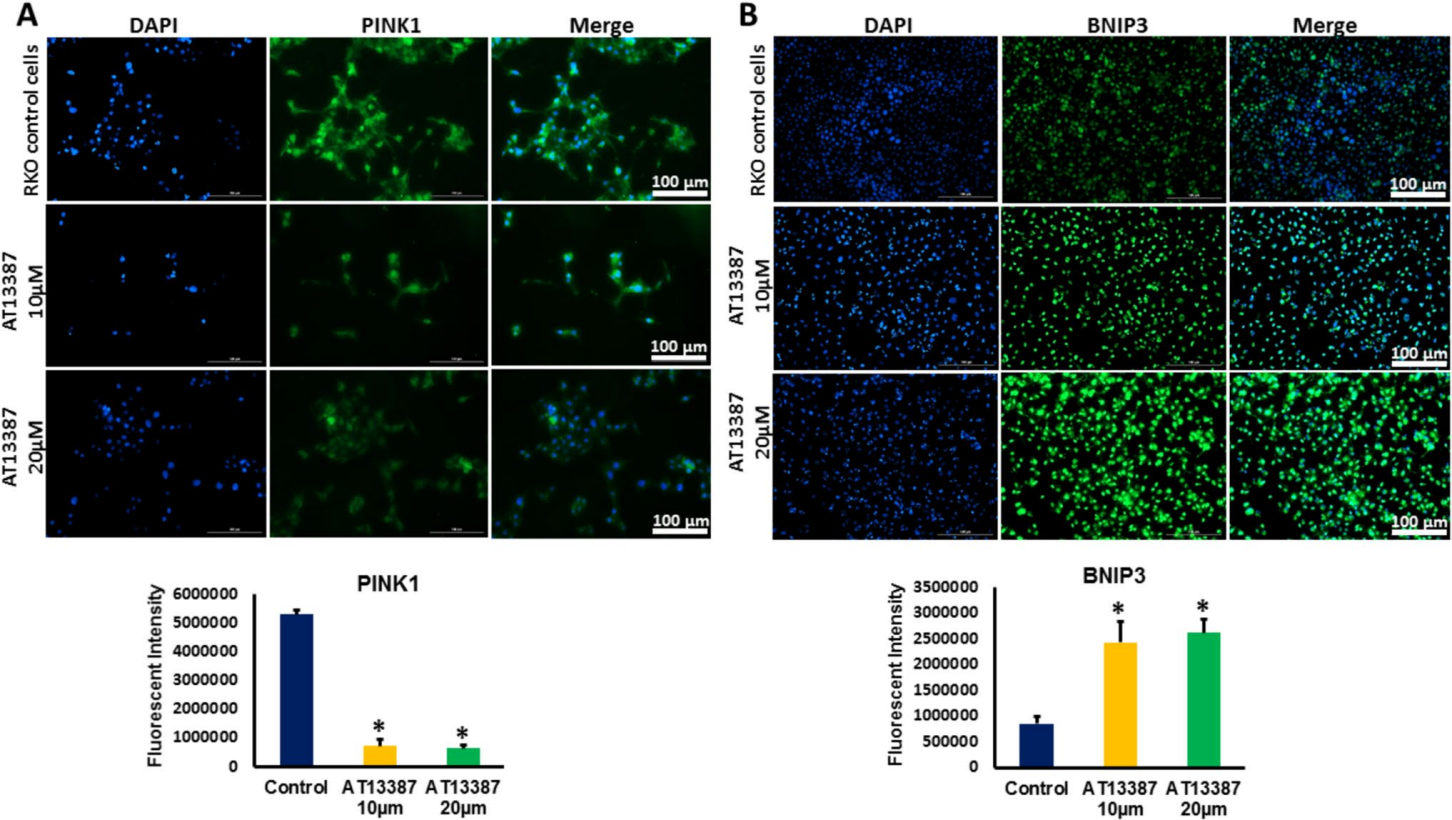
Immunofluorescent analysis of PINK1 and BNIP3 in AT13387-treated RKO cells. **A**The expression of PINK1(green fluorescent color) significantly declined in RKO cells treated with the two doses of AT13387 (10 and 20 μm) compared to untreated cells. **B** The expression of BNIP3 (green fluorescent color) showed a significant increase in RKO cells treated with the two doses of AT13387 compared to untreated cells. N = 3, *P < 0.05 is statistically significant compared to control group

To investigate the effect of HSP90 inhibition on mitochondrial function in RKO cells, we first evaluated the effect of HSP90 inhibition on the expression of PINK1, a mitochondria-targeted serine/threonine kinase that is functionally related to mitochondrial quality control by playing a key protective in preserving mitochondrial integrity and preventing oxidative stress and apoptosis [15]. Our results showed that the expression of PINK1 was significantly decreased in RKO cells treated with 10 µm and 20 µm AT13387 inhibitor compared to control cells (Figs. 3A, B and Fig. 4A). These results indicate that HSP90 inhibition can disturb the mitochondrial quality control process and increase the tendency to trigger oxidative stress in RKO cells. In order to correlate the loss of mitochondrial guardian; PINK1 with incitement of oxidative stress, we evaluated the expression of Bcl-2 and BNIP3. The inducible expression of BNIP3 in the mitochondria elicits the loss of mitochondrial membrane potential and increases the production of reactive oxygen species triggering cell death signaling pathways [16]. Our results demonstrated that HSP90 inhibition induced significant upregulation in BNIP3 protein level in in RKO cells (Figs. 3A, C and Fig. 4B).

### Inhibiting HSP90 instigated BNIP3-mediated oxidative stress in RKO cells

**Fig. 5:**
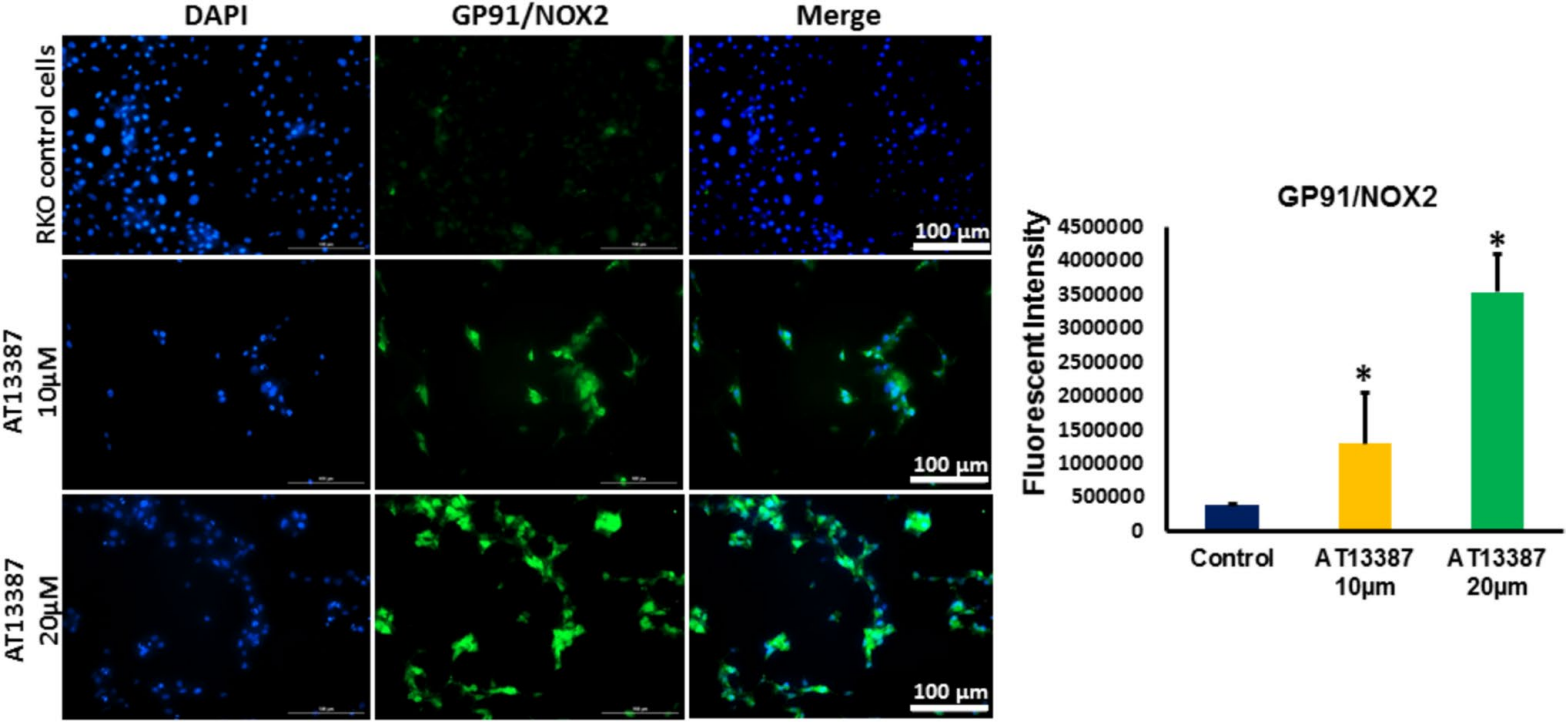
Immunofluorescent analysis of GP91/NOX2 in AT13387-treated RKO cells. The protein level GP91/NOX2 (green fluorescent color) was significantly increased in RKO cells treated with the two doses of AT13387 (10 and 20 μm) compared to untreated cells. N = 3, *P < 0.05 is statistically significant compared to control group

Given the results reported above, which highlighted the potential decrease in the expression of oxidative stress damper; PINK1 and the increase in the expression of oxidative stress inciter; BNIP3 in RKO cells treated with AT13387inhibitor, we measured the expression of oxidative stress marker GP91. Our results demonstrate that treatment of RKO cells with AT13387 inhibitor is associated with significant dose-related increase in the expression of GP91 in RKO cells (Figs. 3A,D and Fig. 5). Furthermore, our findings revealed that inhibiting HSP90 is also linked with increased level of endoplasmic reticulum (ER) stress mediated by remarkable increase in the expression of ER stress sensor, the inositol-requiring transmembrane kinase/endoribonuclease 1α (IRE1α) and the downstream marker of intense ER stress; the protein kinase R-like endoplasmic reticulum kinase (PERK) in RKO cells treated with AT13387 compared to control cells (Figs. 3A,E,F). The elevation in the level of stress markers; GP91 and IRE1α is strongly related to the loss of PINK1 and elevation in BNIP3 level.

### The Pharmacological inhibition of HSP90 in RKO cells was confirmed by genetic knockdown of HSP90 using siRNA

**Fig. 6:**
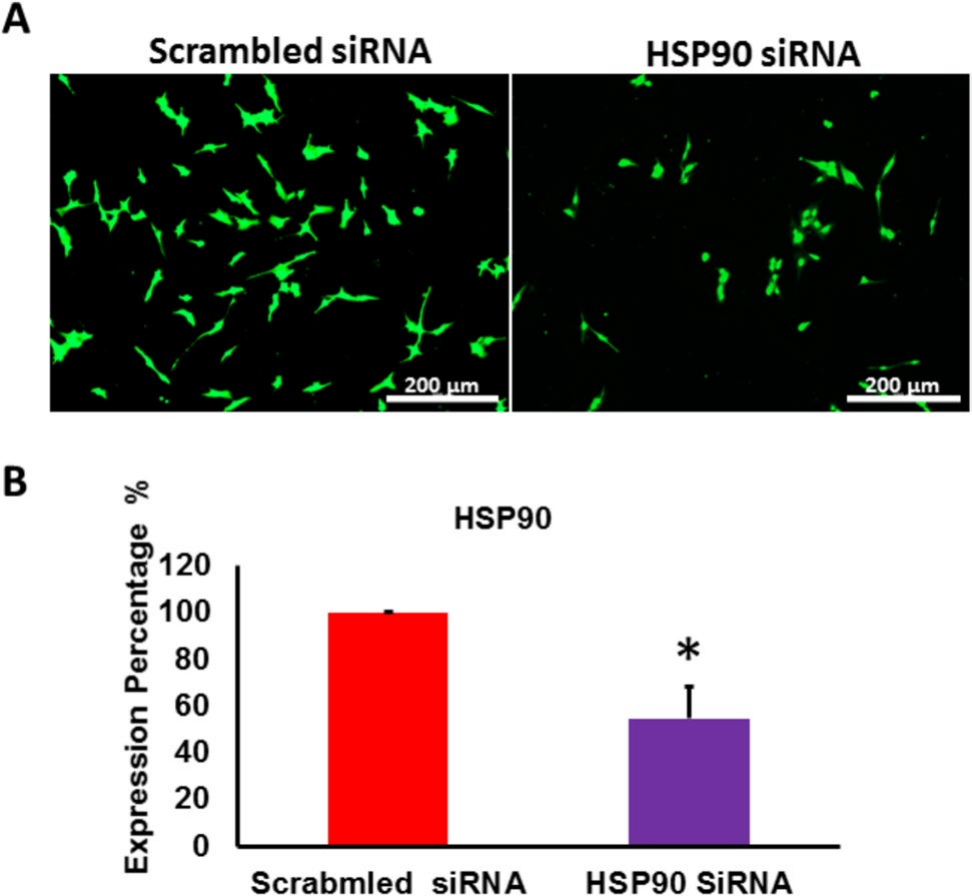
Genetic suppression of HSP90 with siRNA successfully reduced HSP90 levels in RKO cells. **A** Representative images display HSP90 immunofluorescence in RKO cells treated with both targeting and non-targeting siRNA. **B** Immunofluorescence analysis showed a significant reduction (roughly 50%) in HSP90 expression in RKO cells after being transfected with the targeting siRNA compared to scrambled control group. (*p < 0.05 vs. control, n = 3)

The pharmacological inhibition of HSP90 in RKO cells was confirmed through genetic knockdown of HSP90. Knocking down HSP90 by siRNA was initiated in RKO cells. The expression of HSP90 was reduced significantly in transfected RKO cells with targeting siRNA (around 50% reduction in HSP90 expression (Figs. 6A, B).

**Fig. 7:**
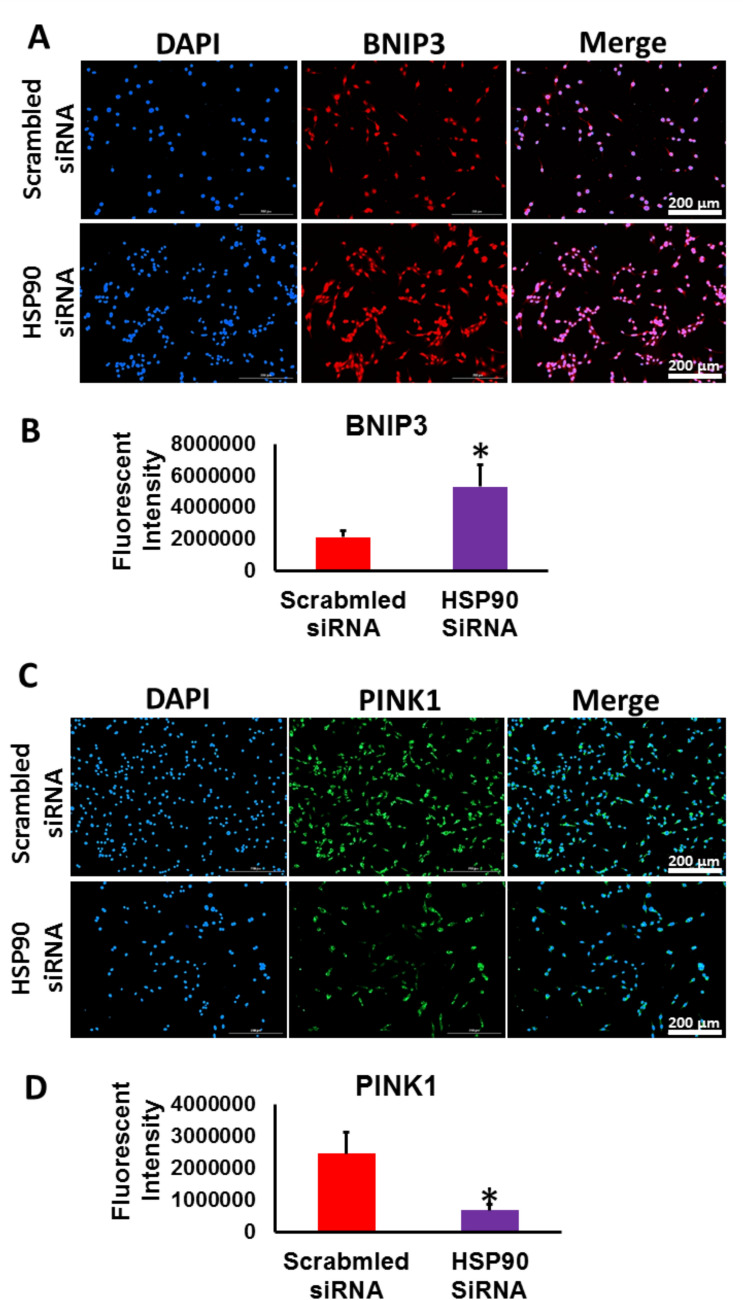
HSP90 -KD RKO cells demonstrated a significant upregulation in BNIP3 level and a down regulation in PINK1 level. **A** Representative images showed BNIP3 immunofluorescence in HSP90-KD RKO cells and control RKO cells. **B** The fluorescent intensity analysis revealed a significant increase in BNIP3 expression following HSP90 knockdown in RKO cells compared to scrambled control. **C** Representative images show PINK1 immunofluorescence in HSP90-KD RKO cells and control cells **D** The fluorescent intensity analysis revealed a significant decrease in pink1 expression following HSP90 knockdown in RKO cells compared to scrambled control. (*p < 0.05 vs. control, n = 3)

To validate the results obtained after inhibiting HSP90 in RKO cells using AT13387, we examined the levels of BNIP3 and PINK1 in HSP90- KD RKO cells. In (Figs. 7A, B), BNIP3 expression was significantly upregulated, while PINK1 expression was significantly downregulated (Figs. 7C, D) in JHSP90-KD RKO cells compared to scrambled control cells. These findings mirrored those from RKO cells treated with the AT13387 inhibitor, reinforcing the relationship between HSP90 inhibition and the modulation of PINK1 and BNIP3 levels.

**Fig. 8:**
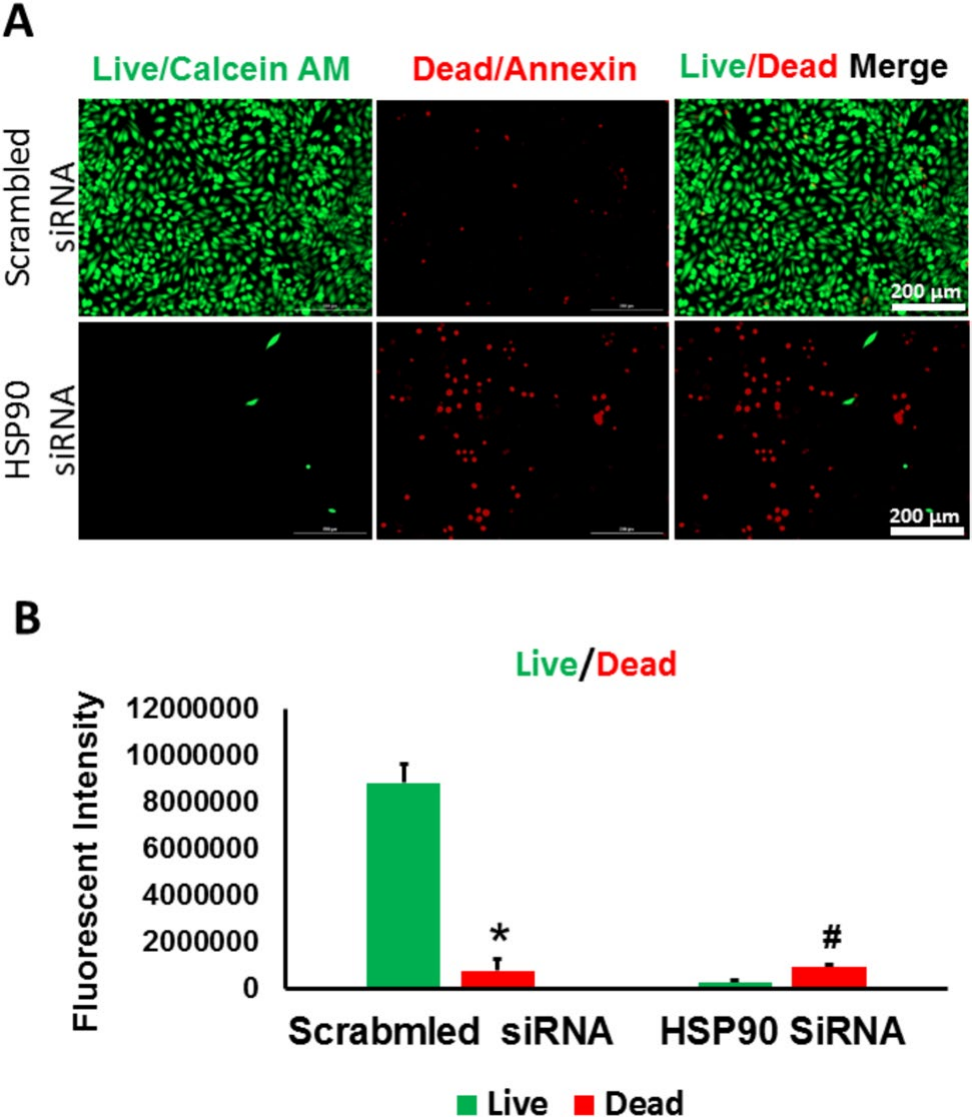
knocking down HSP90 in RKO cells reduced the survival rate and increased the death rate in RKO cells. **A** Representative fluorescent images after performing the LIVE/DEAD in HSP90-KD RKO cells and control cells. **B** fluorescent intensity analysis showed a significant decrease in the live cell percentage and a notable increase in dead cell percentage after knocking down HSP90 in RKO cells compared to scrambled control. (*p < 0.05 vs. control, n = 3)

We subsequently assessed the impact of knocking down HSP90 on the viability of RKO cells by evaluating the level of live and dead cells. Our results demonstrated that HSP90- KD RKO cells showed a significant decrease in the number of live cells and a corresponding significant increase in the number of dead cells (Figs. 8A, B) indicating that knocking down HSP90 in RKO cells induced apoptosis in RKO cells as a result of modulating PINK1 and BNIP3 levels.

## Discussion

Treatment of colon cancer is considered challenging, as many colon cancer cases exhibit poor response to treatment regimen, particularly recurrent types of colon cancer. During the last decade, various strategies have been applied or investigated to improve the treatment protocols for resistant forms of colon cancer [17–19]. Finding new therapies for these resistant and recurrent types of colon cancer is crucial to improving the survival rate of suffering patients. Colon cancer cells are similar to other types of cancer cells by having the ability to amplify many molecular pathways that are essential to promote their proliferation, metastasis, and tumorigenesis [20]. Breaking the balance between cell proliferation and cell death is fundamental to treat aggressive forms of cancer. HSP90 chaperone is the mastermind in regulating many cellular mechanisms to promote the survival and tolerance of cancer cells [21]. HSP90 is integral for stabilizing many anti-apoptotic factors and oncogenic kinases, while silencing apoptosis-related markers [21]. Referring to the importance of HSP90 in providing cancer cells with the fuel needed to support their survival, cancer cells have a higher tendency to overexpress HSP90 [22]. The multifunctional roles of HSP90 in cancer cells have rendered this chaperone as a valuable target for research. We analyzed the TCGA database and identified reports indicating potential mutations and differential expression of PINK1 and HSP90 across various cancer types, including leukemia, adenocarcinomas, and epithelial cell neoplasms. Furthermore, we observed that the expression profiles of these genes differ between normal and cancerous tissues, offering valuable insights into the role of HSP90 in tumorigenesis.

Understanding the downstream molecular basis of HSP90 inhibition can help in developing a more targeted therapy for colon cancer.

Rakitina and co-authors reported that HSP90 inhibitors were effective in enhancing oxaliplatin-dependent caspase activation and cytotoxicity by down-regulating anti-apoptotic signaling and activating NF-κB in colon cancer cells [23]. Moser and co-authors also reported that HSP90 inhibitor;17-DMAG, enhanced the growth inhibitory and pro-apoptotic effects of oxaliplatin in p53-deficient colon cancer cells [24]. The outcomes of these studies are consistent with our study findings as we reported that the pharmacological or genetic blocking of HSP90 in RKO cancer cells; a poorly differentiated and rapidly growing colon cancer cells, induced intense apoptosis and reduced their viability. The inhibition of HSP90 can instigate significant cellular changes and break the infinite cycle of proliferation in colon cancer cells [25, 26]. Identifying the most sensitive and quick acting factors is crucial to understand the molecular basis of HSP90 inhibition. PINK1 is known for being an important guardian for preserving mitochondrial integrity and dynamics, thus it plays an essential role in mitochondrial quality control [27]. The lack of PINK1 found to induce massive mitochondrial dysfunction and apoptosis [28]. There is no clear correlation between HSP90 and PINK1. Herein, we revealed for the first time that the inhibition of HSP90 in colon cancer cells was associated with the downregulation in PINK1, thus terminating its protective roles. PINK1 can initiate many defensive mechanisms to restore the normal mitochondrial functions and prevent oxidative stress which is essential for prolonging the survival of cancer cells. Downregulating PINK1 ensued after inhibiting HSP90 incited intense oxidative stress in colon cancer cells. We found that the loss of PINK1 after inhibiting HSP90 triggered the activation of BNIP3 to ignite the oxidative stress pathway. It was reported initially that slight upregulation in BNIP3 can activate a protective form of autophagy in cancer cells [29]. The sharp elevation in BNIP3 following the inhibition of HSP90 favored the activation of apoptosis cascade. Besides triggering apoptosis, BNIP3 can initiate other types of death pathways, including endoplasmic reticulum stress [30]. We reported in our study that as a result of BNIP3 activation in AT13387 –treated cells, the levels of GP91/NOX2; an oxidative stress marker, and IRE1α; an endoplasmic reticulum stress factor, were significantly increased.

These findings demonstrated that HSP90 inhibition can activate many death pathways as a result of downregulating PINK1 and upregulating BNIP3. By enkindling multiple death signaling pathways, HSP90 inhibition can be considered as an effective therapeutic approach for treating resistant and non-responsive types of colon cancer cells. Qiu and co-authors reported that combining HSP90 inhibitors; anespimycin and ganetespib, with piperlongumine was therapeutically effective in inducing massive endoplasmic reticulum stress and cell cycle arrest in colon cancer cells [31]. Zhang and co-authors found that overexpressing BNIP3 in dopaminergic cells can trigger oxidative stress and endoplasmic reticulum stress as it can dually localize to the mitochondria and endoplasmic reticulum, however these findings haven’t been confirmed in cancer cells (32). Our study added to the growing body of evidence by supporting the efficacy of HSP90 inhibition as a potential therapeutic measure to treat cancer. We provided strong evidence that inhibiting HSP90 is considered enhances multiple death signaling pathways mediated by targeting PINK1- BNIP3 axis. The findings of our study is clinically relevant as it provided an insightful molecular leads that can be targeted in future drug discovery studies. Future studies should validate the reported mechanistic effects and the doses of AT13387 inhibitor in treating other forms of colon cancers and other types of cancer cells.

## Conclusion

We reported for the first time that HSP90 inhibition intensely increased oxidative stress and endoplasmic reticulum stress through downregulating PINK1 and upregulating BNIP3 in colon cancer cells, subsequently reduced colon cancer cells survival and increased its apoptosis. In this context, understanding the therapeutic potential of HSP90 inhibition in curbing cancer progression and tumorigenesis has made HSP90 an effective target for cancer therapy and future drug discovery studies. Detailed characterization of the molecular pathways influenced by HSP90 inhibition may improve its targeted use.

## Supplementary Information

Below is the link to the electronic supplementary material.

Supplementary file1 (DOCX 516 KB)

## Data Availability

No datasets were generated or analysed during the current study.
