# Supplementary material for: Inhibiting HSP90 changes the expression pattern of PINK1 and BNIP3 and induces oxidative stress in colon cancer cells
Source: Mol Biol Rep. 2025 Feb 8;52(1):212. doi: 10.1007/s11033-025-10303-x (PMC11807072; doi:10.1007/s11033-025-10303-x)
Supplement: Supplementary file 1 — Supplementary file1 (DOCX 516 KB) [file 11033_2025_10303_MOESM1_ESM.docx]

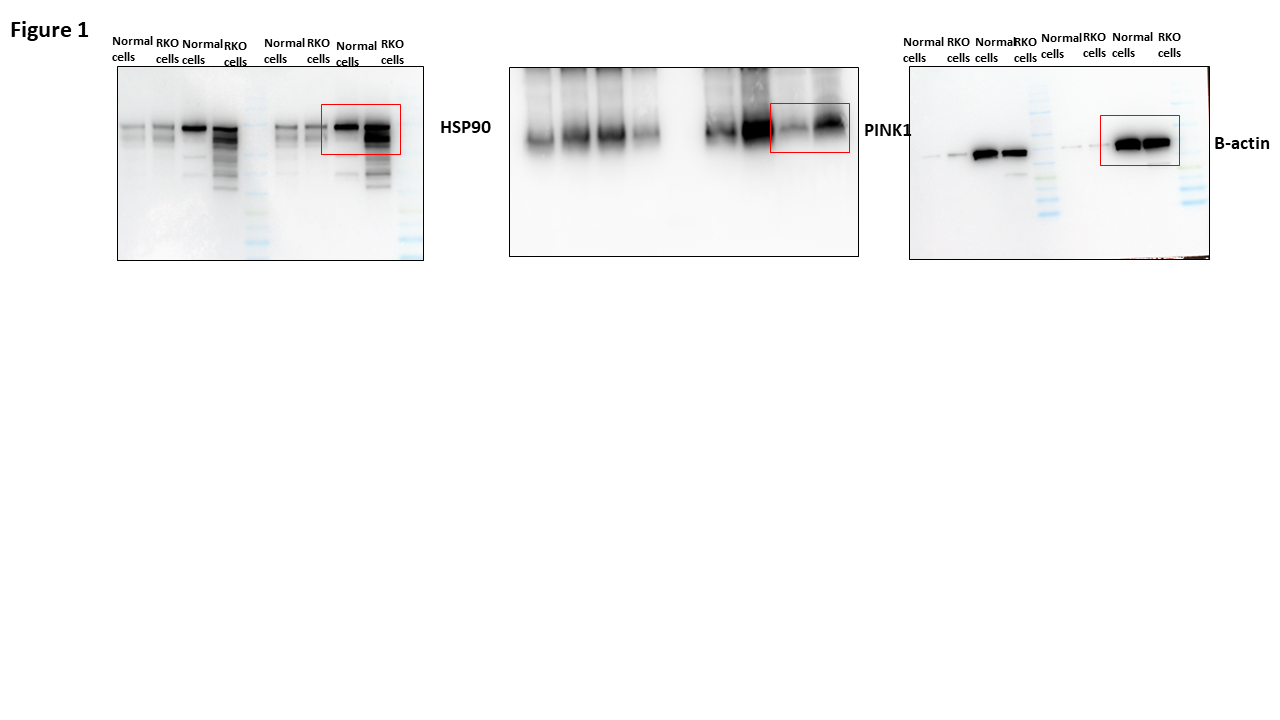


Supplementary Figure 1: Uncropped original Western blots used in Figure 1.


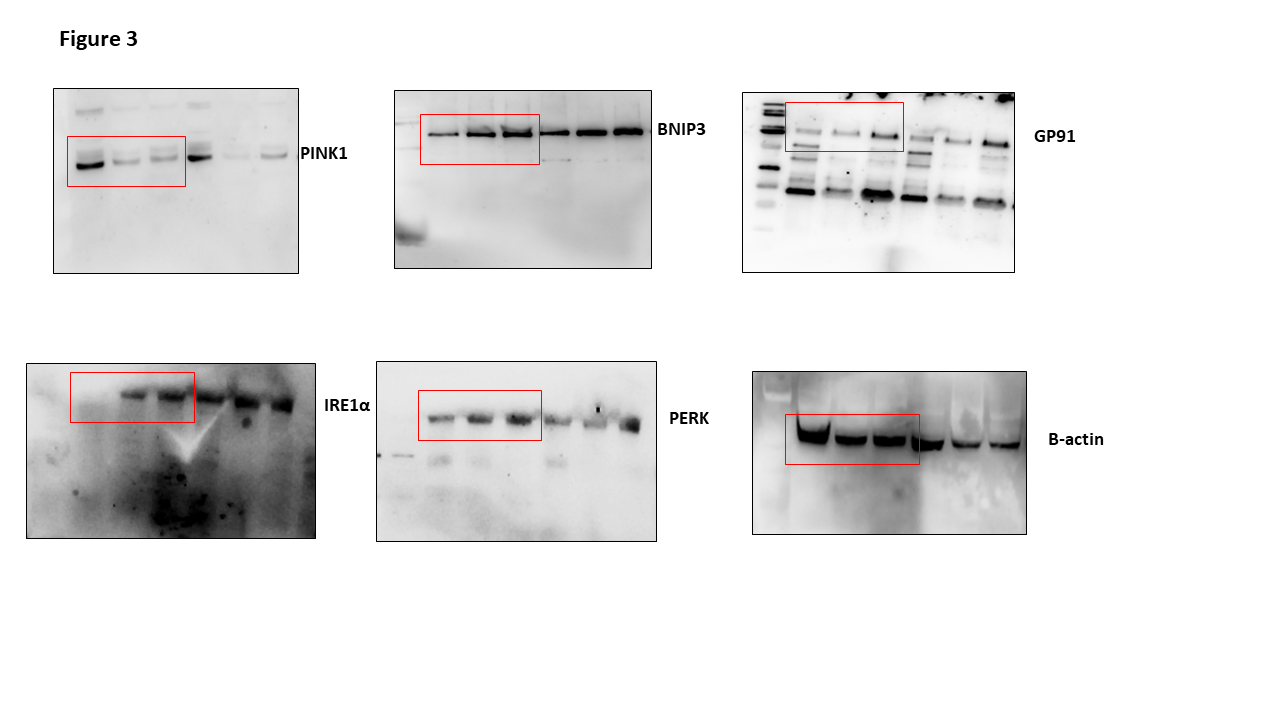


Supplementary Figure 2: Uncropped original Western blots used in Figure 3.
